# Echocardiographic and pathologic correlates of a papillary muscle abscess caused by Neosartorya pseudofischeri

**DOI:** 10.1016/j.idcr.2022.e01504

**Published:** 2022-04-14

**Authors:** Emelyn Zaworski, Andrew Sepiol, FNU Shweta, Andrew D. Calvin

**Affiliations:** a Medical College of Wisconsin – Central Wisconsin, Wausau, WI 54401, USA; b Department of Infectious Disease, Mayo Clinic Health System, Eau Claire, WI 54703, USA; c Department of Cardiovascular Medicine, Mayo Clinic Health System, Eau Claire, WI 54703, USA

**Keywords:** Neosartorya pseudofischeri, Aspergillus fumigatus, Papillary muscle abscess, infective endocarditis, Immunosuppressed host

A 36-year-old Caucasian woman [1] presented to the echocardiography laboratory for evaluation of pulmonary edema in the setting of persistent right lower lung abscess. Past medical history was notable for cystic fibrosis status-post bilateral lung transplant complicated by chronic rejection on high-dose immunosuppressive therapy with tacrolimus, azathioprine, and prednisone. Bronchoalveolar lavage had grown multiple colonies morphologically identified as *A. fumigatus.* Anti-fungal treatment with posaconazole was initiated then broadened to include amphotericin B and caspofungin due to clinical deterioration. Computed tomography (CT)-guided transthoracic lung biopsy from the abscess was sent for molecular sequencing and the isolate was identified as *Neosartorya pseudofischeri*. Prior isolates that had previously be classified based on morphologic criteria as *A. fumigatus* were then sequenced and relabeled as *N. pseudofischeri* [1].

Echocardiography revealed normal right ventricle size, function was borderline reduced, and estimated right ventricular systolic pressure was 69 mmHg (systolic blood pressure 96 mmHg). The left ventricular chamber size was normal, ejection fraction was 75%. The mitral chordae appeared to have four large ovoid masses (Fig. 1). There was mitral valve prolapse versus flail of the posterior leaflet and significant mitral regurgitation (Fig. 2). Given the normal left ventricular chamber size and hyperdynamic function along with acute pulmonary edema, it was felt that this represented severe acute mitral valve regurgitation. A clinical diagnosis of fungal infective endocarditis was rendered, and over the next 48 h the patient shifted to comfort measures only and expired. Post-mortem examination of the heart showed mitral valve prolapse of the P2 and P3 segments and multiple vegetations on the tips of the papillary muscles and posterior leaflet of the mitral valve measuring 0.8–1.3 cm (Fig. 3) with evidence of fungal colonization on hematoxylin and eosin and Grocott's methenamine silver staining. Gross pathology suggested that this was a tissue invasive myocardial infection caused by hematogenous spread through the capillaries as opposed to contiguous spread from the endocardium into the papillary muscle.

**Fig. 1:**
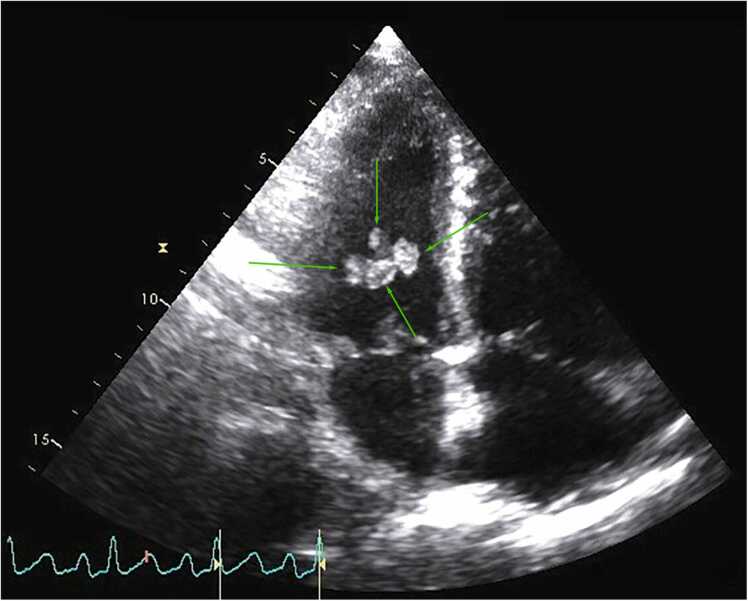
Apical four chamber view with arrows pointing to a four ovoid masses on the mitral chordae. Video clip available.

**Fig. 2:**
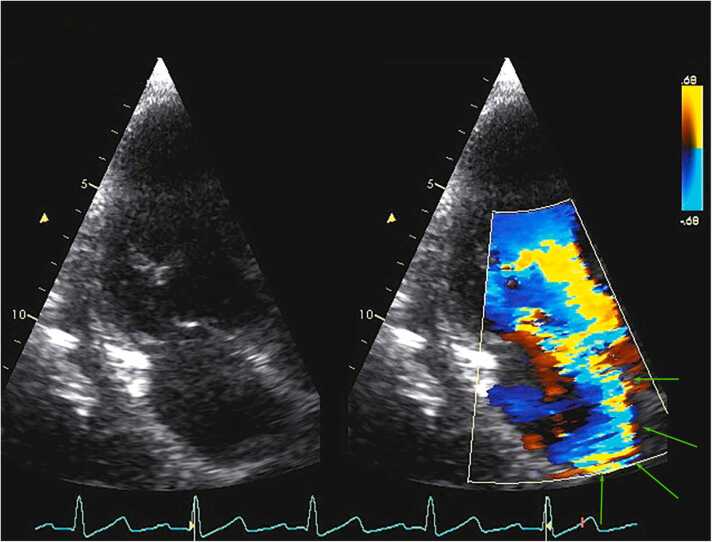
Apical long-axis view showing severe prolapse versus flail posterior mitral leaflet and ovoid masses on the left. On the right is a color flow image showing an eccentric, wall-hugging jet of severe mitral valve regurgitation in systole. This wall-hugging pattern of regurgitation represents the Coanda effect, indicated by arrows. This effect may cause regurgitation to appear less severe due to loss of energy as the jet curves around the wall of the atrium and with less blood to entrain round the high-velocity jet [4].

**Fig. 3:**
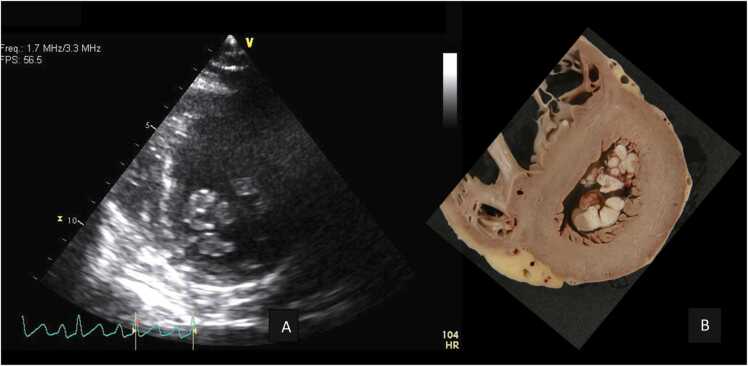
Left (A), echocardiographic apical short axis view demonstrating multiple masses on the chordae tendinea, compared with gross specimen oriented similarly showing vegetation on the tips of the papillary muscles on the right (B).

Fungal infection is a relatively rare cause of endocarditis and is typically only seen in immunosuppressed or immunocompromised patients. Echocardiographic features to distinguish fungal versus bacterial endocarditis are not well described, although some have suggested that bulky lesions suggest a fungal cause [2], [3]. Correlation with microbial data is key to the diagnosis of fungal infective endocarditis. This report expands the information presented in Reference 1, the only known case of native valve *N. pseudofischeri* cardiac infection, which allowed for correlation of clinical history, detailed genetic microbiology, echocardiography, and gross pathologic examination.

## Consent

Patient signed a standard Mayo Clinic research authorization.

## Disclosures

No funding.

## CRediT authorship contribution statement

**Emelyn Zaworski**, BS – Drafting and revision of the article. **Andrew Sepiol**, BSN – Writing – Drafting and revision of the article. **Fnu Shweta**, MBBS – Writing – Drafting and revision of the article. **Andrew Calvin**, MD, MPH – Conception, acquisition and analysis of data, drafting and revision of the article.

## Declaration of Competing Interest

None.
